# Genome wide analysis and clinical correlation of chromosomal and transcriptional mutations in cancers of the biliary tract

**DOI:** 10.1186/1756-9966-28-62

**Published:** 2009-05-12

**Authors:** George Miller, Nicholas D Socci, Deepti Dhall, Michael D'Angelica, Ronald P DeMatteo, Peter J Allen, Bhuvanesh Singh, Yuman Fong, Leslie H Blumgart, David S Klimstra, William R Jarnagin

**Affiliations:** 1 Department of Surgery and Cell Biology, New York University School of Medicine, 550 First Avenue, New York, NY 10016, USA; 2 Department of Biostatistics, Memorial Sloan-Kettering Cancer Center, 1275 York Avenue, New York, NY 1002, USA; 3 Department of Pathology, Memorial Sloan-Kettering Cancer Center, 1275 York Avenue, New York, NY 1002, USA; 4 Department of Surgery, Memorial Sloan-Kettering Cancer Center, 1275 York Avenue, New York, NY 10021, USA

## Abstract

**Background:**

The pathogenesis of biliary cancers is ill-defined. This study investigates changes in gene expression and copy number in biliary cancers and correlates these changes with anatomical site of origin, histopathology and outcome.

**Methods:**

We performed gene expression and CGH analysis on 34 biliary tract cancer specimens. Results were confirmed by RT-PCR. Clinical-pathologic correlation was made using functional over-representation analysis of the top 100 mutations associated with each variable.

**Results:**

There were 545 genes with altered expression in extrahepatic cholangiocarcinoma, 2,354 in intrahepatic cholangiocarcinoma, and 1,281 in gallbladder cancer. Unsupervised hierarchical clustering analysis indicated there was no difference in the global gene expression patterns between each biliary cancer subgroup. CGH analysis revealed that short segments of chromosomes 1p, 3p, 6q, 8p, 9p, and 14q were commonly deleted across all cancer subtypes. Commonly amplified regions included segments of 1q, 3q, 5p, 7p, 7q, 8q, and 20q. Over-representation analysis revealed an association between altered expression of functional gene groupings and pathologic features.

**Conclusion:**

This study defined regions of the genome associated with changes in DNA copy number and gene expression in specific subtypes of biliary cancers. The findings have implications for identification of therapeutic targets, screening, and prognostication.

## Background

Biliary tract cancers account for approximately 10–20% of hepatobiliary neoplasms. Approximately 9,000 cases of biliary tumors are diagnosed in the USA each year. Gallbladder carcinoma (GBC) is the most common, accounting for 60% of cases [1]. The remaining 40% are cholangiocarcinomas and are further sub-classified as intrahepatic (IHC) when they arise from intrahepatic biliary radicles or extrahepatic (EHC) when they arise from the confluence of the main left and right hepatic ducts or distal in the bile ducts. The classification of biliary tract cancers into these anatomically-based subtypes has substantial clinical relevance, as risk factors, presentation, staging, and treatment varies for each [2,3]. Regardless of subtype, most patients with carcinoma of the biliary tract present with advanced disease, with median survival of approximately one to two years from the time of diagnosis [4-6].

Little is known regarding the genetic alterations in the biliary epithelium that lead to cancer. Studies have shown that biliary carcinogenesis may be related in-part to loss of heterozygosity at the loci of chromosomes 1p, 6q, 9p, 16q, and 17p, and point mutations at the *K-ras *oncogene and the p-53 tumor suppressor gene [7,8]. Enhanced expression of VEGF in cholangiocarcinoma cells and localization of VEGF receptor-1 and receptor-2 in endothelial cells is thought to play a crucial role in tumor progression [9]. Clyclooxygenase-2 and c-erbB-2 are also overexpressed in cholangiocarcinoma [10]. In addition, interleukin-6 is important in the proliferation of malignant biliary epithelial cells [11,12]. Our recent work examining cell cycle-regulatory protein expression in biliary tract cancers revealed differentially expressed cell cycle-regulatory proteins based on tumor location and morphology, and an overlap in the pathogenesis of GBC and EHC was suggested [13].

The present study investigates alterations in gene expression and gene copy number in frozen tumor specimens from patients with GBC, IHC, and EHC. Gene expression results were correlated with comparative genomic hybridization (CGH) data by identifying transcriptional changes in the most highly unstable genomic regions. Additionally, the genetic findings were correlated with clinical disease characteristics and pathologic features.

## Methods

### Patients and specimens

Biliary tract cancers from 34 patients (13 IHC, 12 EHC, 9 GBC) were snap-frozen and stored at -80°C. In addition 9 non-cancerous gallbladders and 9 non-cancerous bile duct controls were obtained from patients who had resections for diseases not involving the gallbladder or bile duct (in these patients the gallbladder or bile duct was removed for surgical access to other hepatobiliary or pancreatic structures). Each sample was re-examined histologically using H&E-stained cryostat sections. Surrounding non-neoplastic tissue was dissected from the frozen block under 10× magnification and care was taken that at least 90% for remaining cells were cancerous. All studies were approved by the Memorial Sloan-Kettering IRB.

### RNA isolation, probe preparation, and expression microarray hybridization

Total RNA was isolated from tissue using the DNA/RNA all prep kit (Qiagen, Germantown, Maryland, USA). Quality of RNA was ensured before labeling by analyzing 20–50 ng of each sample using the RNA 6000 NanoAssay and a Bioanalyzer 2100 (Agilent, Santa Clara, California, USA). Samples with a 28S/18S ribosomal peak ratio of 1.8–2.0 and a RIN number >7.0 were considered suitable for labeling. RNA from one IHC specimen, two EHC specimens, and three cases of GBC failed to meet this standard and were discarded from the gene expression analysis. For the remaining samples, 2 μg of total RNA was used for cDNA synthesis using an oligo-dT-T7 primer and the SuperScript Double-Stranded cDNA Synthesis Kit (Invitrogen, Carlsbad, California, USA). Synthesis, linear amplification, and labeling of cRNA were accomplished by in-vitro transcription using the MessageAmp aRNA Kit (Ambion, Austin, Texas, USA) and biotinylated nucleotides (Enzo Diagnostics, New York, USA). Ten micrograms of labeled and fragmented cRNA were then hybridized to the Human HG-U133A GeneChip (Affymetrix, Santa Clara, California, USA) at 45°C for 16 hours. Post hybridization staining, washing were processed according to manufacturer. Finally, chips were scanned with a high-numerical aperture and flying objective lens in the GS3000 scanner (Affymetrix). The image was quantified using GeneChip Operating Software (GCOS) 1.4 (Affymetrix).

### Array CGH profiling

Genomic DNA was extracted using the DNA/RNA prep kit (Qiagen). DNA integrity was checked on a 1% agarose gel and was intact in all specimens except one case of EHC. 3 μg of DNA was then digested and labeled by random priming using RadPrime (Invitrogen) and Cy3 or Cy5-dUTP. Labeled DNA was hybridized to 244 K CGH arrays (Agilent) for 40 hours at 60°C. Slides were scanned and images quantified using Feature Extraction 9.1 (Agilent).

### Real-Time PCR

1 ug of total RNA was reverse-transcribed using the Thermoscript RT-PCR system (Invitrogen) at 52°C for 1 h. 20 ng of resultant cDNA was used in a Q-PCR reaction using an iCycler (Biorad, Hercules, California, USA) and pre-designed TaqMan ABI Gene expression Assays (Hs00270424_m1 for *CCNB2*, Hs00938777_m1 for *CDC2*, Hs00175938_m1 for *CDKN1C*, Hs01665258_m1 for *DLC1*, Hs01547109_m1 for *FOSB*, Hs99999032_m1 for *IL6*, s01118813_m1 for *NR4A2*, Hs00971643_g1 for *SRD5A1*, Hs01014001_m1 for *STAT1*, Hs00426591_m1 for *TYMS*, Hs00197374_m1 for *UBD*). Primers were chosen based on their ability to span the most 3' exon-exon junction. Amplification was carried for 40 cycles (95C for 15 sec, 60C for 1 min). To calculate the efficiency of the PCR reaction, and to assess the sensitivity of each assay, we also performed a 7 point standard curve (5, 1.7,0.56,0.19,0.062,0.021, and 0.0069 ng). Amounts of target were interpolated from the standard curves and normalized to *HPRT *(Hs99999909_m1).

### Data Analysis

Image files were quantified using GCOS 1.1 to generate the CEL files. These were normalized using the GC-RMA package from the Bioconductor toolkit (Bioconductor, Seattle, Washington State, USA). Expression values were log (base 2) transformed for all subsequent analysis. Unsupervised hierarchical clustering was done using a distance measure derived from the Pearson correlation (distance = (1-ρ)/2 were ρ is the correlation coefficient) and average linkage options. To determine differentially expressed genes a variant of the t- and F-tests were used as implemented in the LIMMA toolkit (Bioconductor). To account for multiple-testing the False Discovery Rate (FDR) method was used. An FDR < 0.01 was considered statistically significant. For clinicopathologic correlation, a functional over-representation analysis was done on the top 100 genes. p < 0.001 was considered significant.

For the array-CGH data, the raw images were quantified with the Agilent Feature Extraction program and normalized using a combination of intensity dependent and GC-content dependent non-linear normalization procedure. To determine significant changes in copy number, the Circular Binary Segmentation algorithm [14] was used with alpha set to 0.001. Segments that had a log 2 ratio of intensity greater than a sample dependent threshold and a signal-to-noise ratio greater than 0.5 were considered either amplified or deleted.

## Results

### Clinicopathologic Data

Frozen tissue was analyzed from 34 patients who underwent surgery for biliary tract cancers between August 1993 and December 2005. 13 patients had IHC, 12 had EHC, either at the bile duct bifurcation or in the mid or distal bile duct, and 9 patients had tumors originating within the gallbladder. Selected clinicopathologic features are shown in Table 1. The median age of patients was 64 (range 46–88) and 20 (59%) patients were female. 31 (91%) patients had margin-negative resections, two (6%) patients had margin-positive resections, and one (3%) patient underwent biopsy only.

**Table 1:** Clinicopathologic features of biliary tract cancer patients in this study

**Biliary Cancer Subtype**	**Age**	**Sex**	**Lymph Node Invasion**	**Vascular Invasion**	**Perineural Invasion**	**Pathologic Differentiation**	**Size (cm)**	**Follow-up (months)**	**Disease Status^*a*^**
Extrahepatic	77	F	Present	Absent	Present	Poor	2.0	42	DOD
Extrahepatic	57	F	Present	Present	Present	Moderate	1.5	61	DOD
Extrahepatic	60	M	Present	Present	Present	Poor	1.6	18	DOD
Extrahepatic	78	M	Absent	Present	Present	Poor	1.7	16	NED
Extrahepatic	81	F	Absent	Absent	Absent	Well	3.1	58	AWD
Extrahepatic	75	M	Absent	Present	Absent	Moderate	2.2	87	AWD
Extrahepatic	77	F	Absent	Absent	Present	Moderate	4.0	45	DOD
Extrahepatic	56	M	Absent	Absent	Present	Moderate	2.0	13	DOD
Extrahepatic	67	F	Absent	Absent	Present	Moderate	1.8	20	DOD
Extrahepatic	56	M	Absent	Present	Present	Moderate	4.8	40	DOD
Extrahepatic	62	M	Absent	Absent	Absent	Well	5.9	58	NED
Extrahepatic	47	M	Absent	Absent	Present	Moderate	2.3	6	DOD
Intrahepatic	64	M	Absent	Absent	Absent	Moderate	8.0	32	DOD
Intrahepatic	66	F	Absent	Present	Absent	Moderate	13.0	6	DOD
Intrahepatic	63	M	Absent	Present	n/a	Poor	9.9	14	DOD
Intrahepatic	56	M	Absent	Present	Absent	Moderate	11.0	18	DOD
Intrahepatic	70	M	Absent	Absent	n/a	Moderate	6.0	98	NED
Intrahepatic	53	F	Absent	Present	Present	Moderate	8.5	23	DOD
Intrahepatic	60	F	Absent	Absent	Absent	Poor	18.0	40	DOD
Intrahepatic	68	F	Absent	Absent	Absent	Moderate	12.0	33	DOD
Intrahepatic	50	M	Absent	Absent	Absent	Well	21.0	68	NED
Intrahepatic	60	F	Absent	Absent	Absent	Moderate	20.0	20	DOD
Intrahepatic	58	M	Present	Present	Absent	Moderate	9.0	38	DOD
Intrahepatic	46	F	Present	Present	Absent	Moderate	7.0	37	NED
Intrahepatic	87	F	Present	Absent	Absent	Moderate	14.0	11	NED
Gallbladder	58	F	Present	Absent	Present	Moderate	1.5	n/a	n/a
Gallbladder	78	F	Absent	Absent	Absent	Moderate	12.0	77	NED
Gallbladder	79	F	Absent	Absent	Absent	Moderate	9.0	62	NED
Gallbladder	51	F	Present	Present	Present	Poor	4.7	24	AWD
Gallbladder	61	F	Present	Present	Present	Moderate	2.0	1	DUC
Gallbladder	88	F	Absent	n/a^*b*^	n/a	Moderate	8.7	2	DOD
Gallbladder	68	F	Absent	n/a	n/a	Moderate	3.5	82	NED
Gallbladder	78	F	Present	Present	Present	Moderate	9.0	3	DOD
Gallbladder	78	M	Present	Present	Present	Moderate	4.7	13	NED

At last follow-up, 10 (29%) patients were alive without evidence of disease, 3 (9%) patients were alive with recurrent disease and 19 (56%) died as a result of their disease. One (3%) patient died of an unrelated cause and one (3%) patient was lost to follow-up. The median follow-up for surviving patients was 58 months (range 11–98). A review of pathologic features revealed that 6 (18%) patients had poorly differentiated tumors, 11 (32%) patients had evidence of lymph node invasion, 15 (44%) had vascular invasion, and 15 (44%) had perineural invasion. The median tumor size was 11.0 cm (range 6.0 – 21.0) for IHC, 2.1 cm (range 1.5 – 5.9) for EHC, and 4.7 cm (range 1.5 – 12.0) for GBC (Table 1).

### Gene Transcriptional Alterations in Biliary Carcinomas

**Figure 1:**
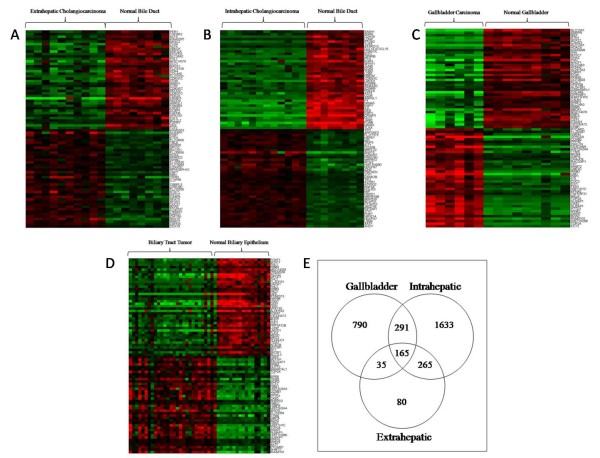
**Gene Expression Alterations in Biliary Tract Cancers**. Heat maps showing the top 40 overexpressed (red) and top 40 underexpressed (green) genes for (a) EHC, (b) IHC, and (c) GBC. (d) All malignant subtypes were also combined for analysis and compared in terms of gene expression with benign bile duct and gallbladder controls. Genes were ranked based on FDR values. (e) A Venn diagram is used to depict the relationship of transcriptional changes among biliary cancer subtypes. There were 165 common genes with significantly altered expression in all three biliary tract cancer subtypes.

We analyzed alterations in gene expression in EHC, IHC, and GBC compared with non-cancerous bile duct or gallbladder controls using the Human Genome U133A GeneChip. Figure 1 depicts the 40 top ranking overexpressed and underexpressed genes for (a) extrahepatic cholangiocarcinoma, (b) IHC, and (c) GBC. Ranking was based on FDR values. Table 2 summarizes the extent of gene expression alterations for each type of biliary tract cancer. In the EHC specimens, differential expression was noted in 545 genes compared with 2,354 in IHC and 1,281 in GBC (See additional files 1, additional file 2, and additional file 3). There was a near equal distribution of overexpressed and underexpressed genes for each tumor type. However, higher fold changes in expression levels were seen more commonly with underexpressed genes. In particular, depending on cancer subtype, 16–22% of genes with decreased expression had greater than 10-fold changes expression levels compared with controls. Conversely, only 2–12% of genes with increased expression had alterations of 10-fold or greater (Table 2).

**Table 2:** Summary of transcription mutations in subtypes of biliary tract carcinoma

	Extrahepatic Cholangiocarcinoma	Intrahepatic Cholangiocarcinoma	Gallbladder Carcinoma
Number of transcriptional changes	545	2354	1281

Increased expression	200	1286	479

Decreased expression	345	1068	802

Increased > 20-fold	3	10	26

Increased > 10-fold	16	31	59

Decreased > 20-fold	22	88	72

Decreased > 10-fold	56	227	174

### Comparative Analysis of Biliary Cancer Subtypes

Unsupervised hierarchical clustering analysis revealed that the three cancer subtypes did not cluster separately, implying that there was no difference in the global gene expression patterns between the biliary cancer subgroups. Figure 1d depicts the top 40 up-regulated and down-regulated genes for all cancers combined versus the 18 control specimens. However, while the individual cancer subtypes did not cluster separately, there was unique differential expression of many genes compared with normal biliary epithelium in each cancer subtypes. The relationship of gene transcriptional changes among the three biliary cancer subtypes is depicted in a Venn diagram (Figure 1e). There was unique altered expression of 1633, 80, and 790 genes in IHC, EHC, and GBC, respectively. Overall, 165 probe sets were commonly differentially expressed in all 3 cancer types (See additional file 4). Selected commonly differentially expressed genes are listed in Table 3.

**Table 3:** Selected genes with significantly altered expression in all three biliary cancer subtypes with the fold-change (Δ) in expression

Δ GB	Δ Intra	Δ Extra	Gene Symbol	Gene Title	Chromosomal Location	Functional Pathway
58	11	11	RRM2	ribonucleotide reductase M2 polypeptide	chr2p25-p24	Nucleotide metabolism
41	6	10	PTTG1	pituitary tumor-transforming 1	chr5q35.1	Cell cycle
26	9	7	TYMS	thymidylate synthetase	chr18p11.32	Nucleotide metabolism
19	4	5	CDC2	Cell division cycle 2, G1 to S and G2 to M	chr10q21.1	Cell cycle
18	4	7	CCNB2	cyclin B2	chr15q22.2	Cell cycle
12	3	4	RACGAP1	Rac GTPase activating protein 1	chr12q13.12	S1P Signaling
6	5	4	SHMT2	serine hydroxymethyltransferase 2 (mitochondrial)	chr12q12-q14	Amino acid metabolism
3	3	3	PPAT	phosphoribosyl pyrophosphate amidotransferase	chr4q12	Purine metabolism
3	3	5	MCM6	MCM6 minichromosome maintenance deficient 6	chr2q21	Cell cycle
3	3	3	GMPS	guanine monphosphate synthetase	chr3q24	Nucleotide metabolism
2	2	2	RPS19	ribosomal protein S19	chr19q13.2	Ribosomal protein
2	3	2	CBX3	chromobox homolog 3	chr7p15.2	Circadian exercise
2	3	2	EIF2AK1	eukaryotic translation initiation factor 2-alpha kinase 1	chr7p22	Translation factor
2	2	2	EPRS	glutamyl-prolyl-tRNA synthetase	chr1q41-q42	Glutamate metabolism
2	2	2	PARP1	poly (ADP-ribose) polymerase family, member 1	chr1q41-q42	Apoptosis
2	2	2	SNRPD2	small nuclear ribonucleoprotein D2 polypeptide 16.5 kDa	chr19q13.2	mRNA processing
-2	-2	-2	UBE2G2	ubiquitin-conjugating enzyme E2G 2 (UBC7 homolog)	chr21q22.3	Proteolysis
-2	-2	-2	HNRPH1	Heterogeneous nuclear ribonucleoprotein H1	chr5q35.3	mRNA processing
-2	-3	-3	SUI1	putative translation initiation factor	chr17q21.2	Translation factor
-3	-4	-3	RBM5	RNA binding motif protein 5	chr3p21.3	mRNA processing
-3	-2	-2	SFRS5	splicing factor, arginine/serine-rich 5	chr14q24	mRNA processing
-3	-3	-3	BCL2L2	BCL2-like 2	chr14q11.2-q12	Apoptosis
-4	-10	-7	CDKN1C	cyclin-dependent kinase inhibitor 1C (p57, Kip2)	chr11p15.5	G1 to S cell cycle
-4	-8	-5	ZNF423	zinc finger protein 423	chr16q12	TGF-β signaling
-4	-3	-3	ACACB	acetyl-Coenzyme A carboxylase beta	chr12q24.11	Fatty acid synthesis
-4	-4	-3	RBM5	RNA binding motif protein 5	chr3p21.3	mRNA processing
-5	-7	-5	PRKAR2B	protein kinase, cAMP-dependent, regulatory, type II, beta	chr7q22	G protein signaling
-5	-4	-4	ACACB	acetyl-Coenzyme A carboxylase beta	chr12q24.11	Fatty acid synthesis
-6	-4	-4	ITGA7	integrin, alpha 7	chr12q13	Cellular adhesion
-6	-7	-5	RGS2	regulator of G-protein signaling 2, 24 kDa	chr1q31	Calcium regulation
-6	-9	-5	KLF9	Kruppel-like factor 9	chr9q13	Circadian exercise
-7	-7	-7	RPS6KA2	ribosomal protein S6 kinase, 90 kDa, polypeptide 2	chr6q27	Ribosomal protein
-7	-15	-10	ANK2	ankyrin 2, neuronal	chr4q25-q27	Ribosomal protein
-8	-5	-6	ACACB	acetyl-Coenzyme A carboxylase beta	chr12q24.11	Fatty acid synthesis
-10	-4	-4	MYOM1	myomesin 1 (skelemin) 185 kDa	chr18p11.32-p11.31	Muscle contraction
-11	-13	-8	ITGA7	integrin, alpha 7	chr12q13	Cellular adhesion
-13	-27	-14	CDKN1C	Cyclin-dependent kinase inhibitor 1C (p57, Kip2)	chr11p15.5	G1 to S cell cycle
-61	-27	-26	ALDH1A2	aldehyde dehydrogenase 1 family, member A2	chr15q21.3	Metabolism/Biosynthesis
-67	-20	-7	CNN1	calponin 1, basic, smooth muscle	chr19p13.2-p13.1	Muscle contraction
-85	-51	-18	CES1	carboxylesterase 1	chr16q13-q22.1	Irinotecan pathway
-102	-30	-24	DES	desmin	chr2q35	Muscle contraction

### Genomic Alterations in Biliary Carcinogenesis

**Figure 2:**
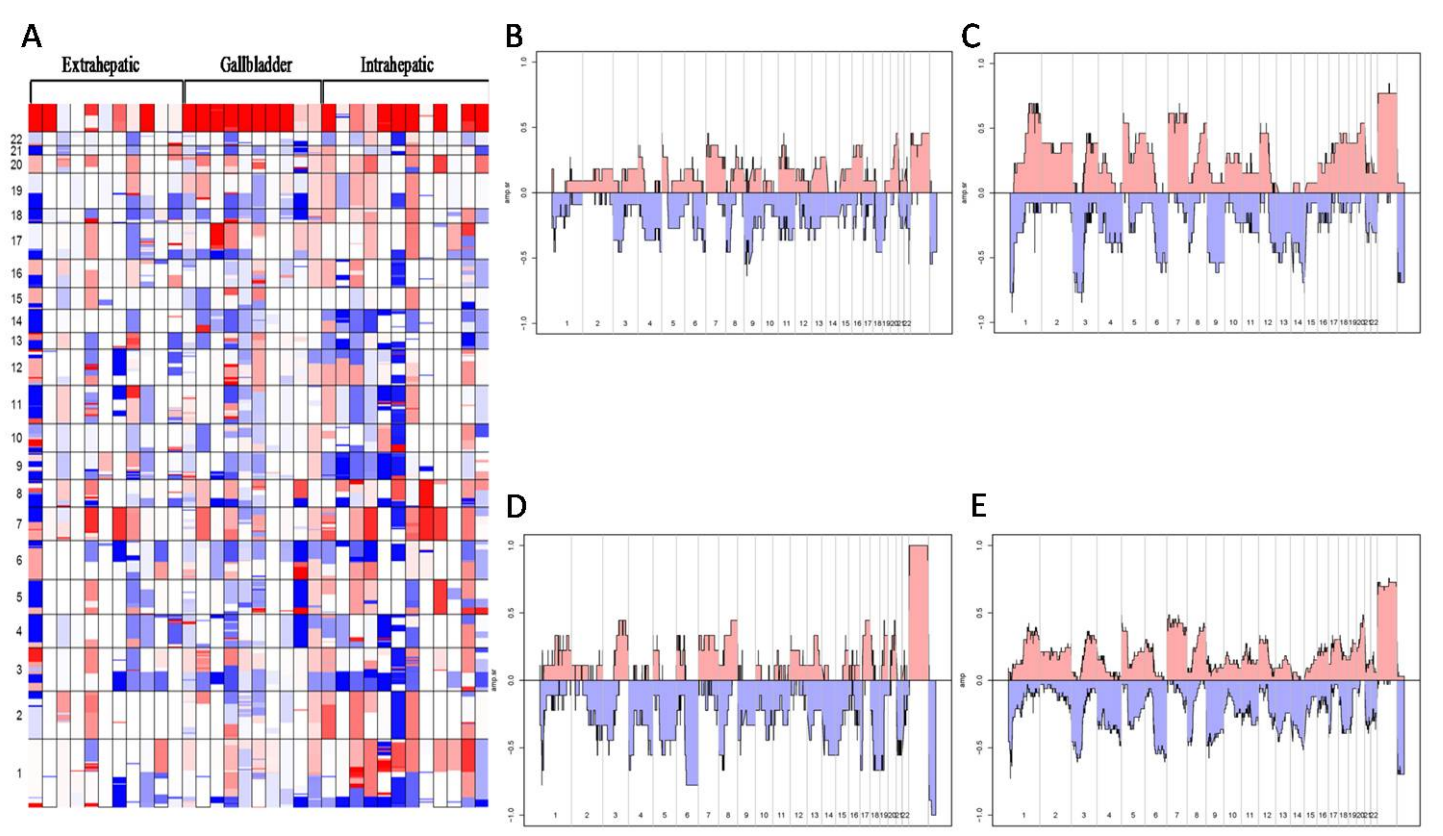
**Chromosomal Structural Mutations in Biliary Tract Cancers**. (a) A cumulative depiction of the copy number changes across the genome for all biliary cancer specimens is shown. Chromosomal number is listed on the left. Amplification is depicted in red and deletion in blue. White is unchanged from genomic DNA controls. Increased amplification or deletion within a cancer specimen is reflected in increased color intensity. The percentage of patient specimens that have either amplifications or deletions at each chromosomal loci is shown for (b) EHC, (c) IHC, (d) GBC, and (e) all biliary tract cancers combined.

To better understand the molecular pathogenesis of biliary tract cancers we used an array based CGH analysis to detect chromosomal areas of DNA copy number gain (DNA copy number of 3 or greater) and loss (DNA copy number of 0 or 1) in the GBC, IHC, and EHC specimens. Figure 2a depicts the chromosomal alterations for each individual cancer specimen while Figure 2b–d represents cumulative summaries of the chromosomal changes for each cancer subtype. Cumulative chromosomal changes for all biliary tract cancers combined are shown in Figure 2e.

Overall, patients with GBC exhibited the greatest genomic instability while patients with IHC had the fewest amplifications and deletions. In particular, the mean number of chromosomal alterations per patient with GBC was 60.6 (range 17–110) with deletions (mean 35.0, range 9–55) more frequent than amplifications (mean 25.6, range 8–55). Patients with IHC had an average of 49.2 alterations (range 11–101) in DNA copy number with slightly more deletions (mean 26.9, range 8–80) than amplifications (mean 22.2, range 2–47). EHC specimens had an average of 43.8 chromosomal alterations (range 3–110) with an average of 22.5 deletions (range 1–61) and 21.4 amplifications (range 1–62). Moreover, there was considerable heterogeneity in the extent of chromosomal instability between patients even within specific cancer subtypes. For example, a number of patients within each cancer subtype had mutations in nearly every chromosomal arm while other patients with the same tumor type had minimal structural changes in their entire genome (Figure 2a).

While the cumulative pattern of chromosomal alterations was highly variable, there appeared to be selected chromosomal regions that were commonly altered across all cancer subtypes. For example, a short segment of chromosome 1p was deleted in greater than 75% of patients with GBC and IHC and nearly 50% of patients with EHC. Similarly, segments of chromosomes 3p, 6q, 8p, 9p, and 14q were commonly deleted across subtypes of biliary cancers. Commonly amplified regions across cancer types include segments of 1q, 3q, 5p, 7p, 7q, 8q, and 20q (Figure 2a–e).

### Analysis of Transcriptional Changes in Commonly Unstable Genomic Regions

To further elucidate the pathogenesis of biliary tract cancers, we integrated the array based CGH data with our gene expression profiling with by identifying gene expression alterations in regions of highest genomic instability. To this end, we investigated the gene expression changes in regions of the genome for which greater than 40% of patients had either chromosomal gains or losses in each cancer subtype (See additional files 5, additional file 6 and additional file 7). Selected alterations in gene expression within these unstable genomic regions are shown in Table 4. Analysis of this data reveals that, as expected, a positive correlation could be made between chromosomal deletion and the loss of gene expression. Conversely, there were no instances of increased gene transcription in regions of chromosomal deletion. However, in regions of chromosomal amplification, both increased and decreased gene transcription were seen with similar frequency.

**Table 4:** Selected changes in gene expression in commonly amplified or deleted regions of the genome for all biliary tract cancer specimens

Chromosomal Location	% Amplified (+) or Deleted (-)	Fold Change	Gene Title	Gene Symbol	Functional Properties
chr7p11	+42%	6.5	IGF-II mRNA-binding protein 3	IMP-3	RNA processing
chr7p13-p12	+45%	3.6	insulin-like growth factor binding protein 3	IGFBP3	Regulation of cell growth
chr5p15.33	+42%	3.5	thyroid hormone receptor interactor 13	TRIP13	Regulation of transcription
chr20q13.32	+45%	3.5	RAE1 RNA export 1 homolog	RAE1	mRNA-nucleus export
chr7p21.1	+48%	3.2	basic leucine zipper and W2 domains 2	BZW2	Translation initiation factor
chr7q22.1	+42%	3.0	origin recognition complex, subunit 5-like	ORC5L	DNA replication initiation
chr20q13.3	+42%	2.7	ribosomal protein S21	RPS21	Protien biosysthesis
chr7p15	+42%	2.6	oxysterol binding protein-like 3	OSBPL3	Steroid metabolism
chr7p15-p13	+42%	2.5	v-ral simian leukemia viral oncogene homolog A	RALA	GTPase mediated signal transduction
chr20q13.2	+48%	-6.9	docking protein 5	DOK5	Insulin receptor binding
chr7q11.2	+42%	-7.8	CD36 antigen	CD36	Lipid metabolism
chr7q21.1	+42%	-7.9	ATP-binding cassette, sub-family B, member 1	ABCB1	Cell surface transport
chr7p21	+45%	-9.1	interleukin 6	IL6	Acute phase response
chr20q11.23	+42%	-10.0	myosin, light polypeptide 9, regulatory	MYL9	Regulation of muscle contraction
chr7q31-q32	+42%	-10.9	solute carrier family 13, member 1	SLC13A1	Ion transport
chr20q13.13	+45%	-14.7	prostaglandin I2 synthase	PTGIS	Prostaglandin biosynthesis
chr7q31	+42%	-38.1	solute carrier family 26, member 3	SLC26A3	Transcription factor activity
chr6q22.1	-55%	-46.2	phospholamban	PLN	Calcium ion transport
chr9q22	-42%	-41.0	osteoglycin	OGN	Growth factor activity
chr6q24-q25	-58%	-19.2	A kinase anchor protein 12	AKAP12	Signal transduction
chr14q24.3	-42%	-17.1	v-fos FBJ murine osteosarcoma viral oncogene homolog	FOS	DNA methylation
chr14q32.1	-45%	-13.6	fibulin 5	FBLN5	Cell-matrix adhesion
chr3p26-p25	-45%	-10.0	inositol 1,4,5-triphosphate receptor, type 1	ITPR1	Cation transport
chr3p13	-52%	-9.2	PDZ domain containing RING finger 3	PDZRN3	Protein ubiquitination
chr3p21.1	-58%	-8.9	TU3A protein	TU3A	Regulation of cell growth
chr14q32.1	-48%	-8.5	serine proteinase inhibitor, clade A, member 5	SERPINA5	Endopeptidase inhibitor
chr3p22-p21.3	-58%	-8.5	C-type lectin domain family 3, member B	CLEC3B	Skeletal development
chr9p13.2-p13.1	-42%	-8.3	tropomyosin 2	TPM2	Muscle development
chr14q32	-48%	-8.1	delta-like 1 homolog	DLK1	Calcium ion binding
chr6q27	-58%	-6.5	ribosomal protein S6 kinase, 90 kDa, polypeptide 2	RPS6KA2	Amino acid phosphorylation
chr6q24-q25	-52%	-6.2	pleiomorphic adenoma gene-like 1	PLAGL1	Regulation of transcription
chr9p13-p12	-42%	-5.8	reversion-inducing-cysteine-rich protein with kazal motifs	RECK	Cell cycle regulation
chr3p21.2-p21.1	-61%	-5.4	aminomethyltransferase	AMT	Glycine catabolism
chr6pter-qter	-48%	-5.4	transcription factor 21	TCF21	Regulation of transcription
chr9q13	-42%	-5.1	Kruppel-like factor 9	KLF9	Regulation of transcription
chr6q23	-48%	-3.8	serum/glucocorticoid regulated kinase	SGK	Amino acid phosphorylation
chr3p26-p25	-45%	-3.6	inositol 1,4,5-triphosphate receptor, type 1	ITPR1	Cell cycle regulation
chr1p36.13-p36.11	-55%	-3.2	neuroblastoma, suppression of tumorigenicity 1	NBL1	calcium ion transport
chr6q22	-55%	-2.6	mannosidase, alpha, class 1A, member 1	MAN1A1	Carbohydrate metabolism
chr3p22	-48%	-2.5	transforming growth factor, beta receptor II	TGFBR2	Regulation of cell proliferation

### Validation of Findings

The Affymetrix U133A gene expression array data were both internally and externally validated. First, a large number of gene transcripts were represented by more than one probe set in the array. In each case, the different probes for each detected similar expression levels of transcript (See additional files 1, additional file 2, and additional file 3). This includes genes with altered expression in EHC (i.e. *CDKN1C, NR4A3, RBM5, SASH1*), IHC (*ADH1B, GREM1, MCM4, NR4A2*), and GBC (*HIST2H2AA, NUSAP1 RPS10, RPS19*).

**Figure 3:**
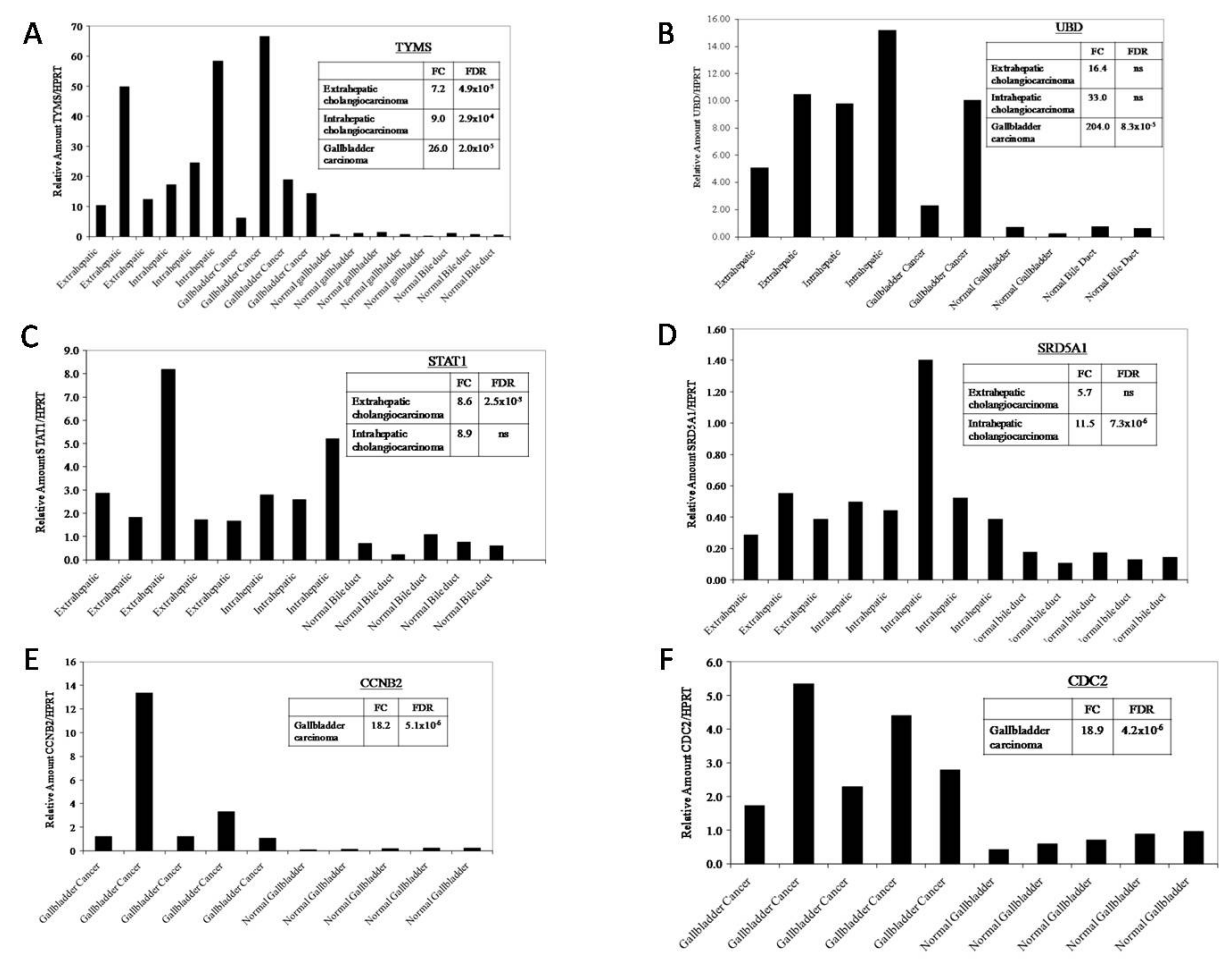
**Real-Time PCR Based Validation of Gene Expression Findings**. To confirm the gene expression changes in biliary tract cancers identified on microarray analysis, selected genes were tested in tumor and control specimens by RT PCR and normalized to *HRPT *which is similarly expressed in tumors and normal biliary epithelia. Results are shown for (a) *TYMS*, (b) *UBD*, (c) *STAT1*, (d) *SRD5A1*, (e) *CCNB2*, (f) *CDC2*.

**Figure 4:**
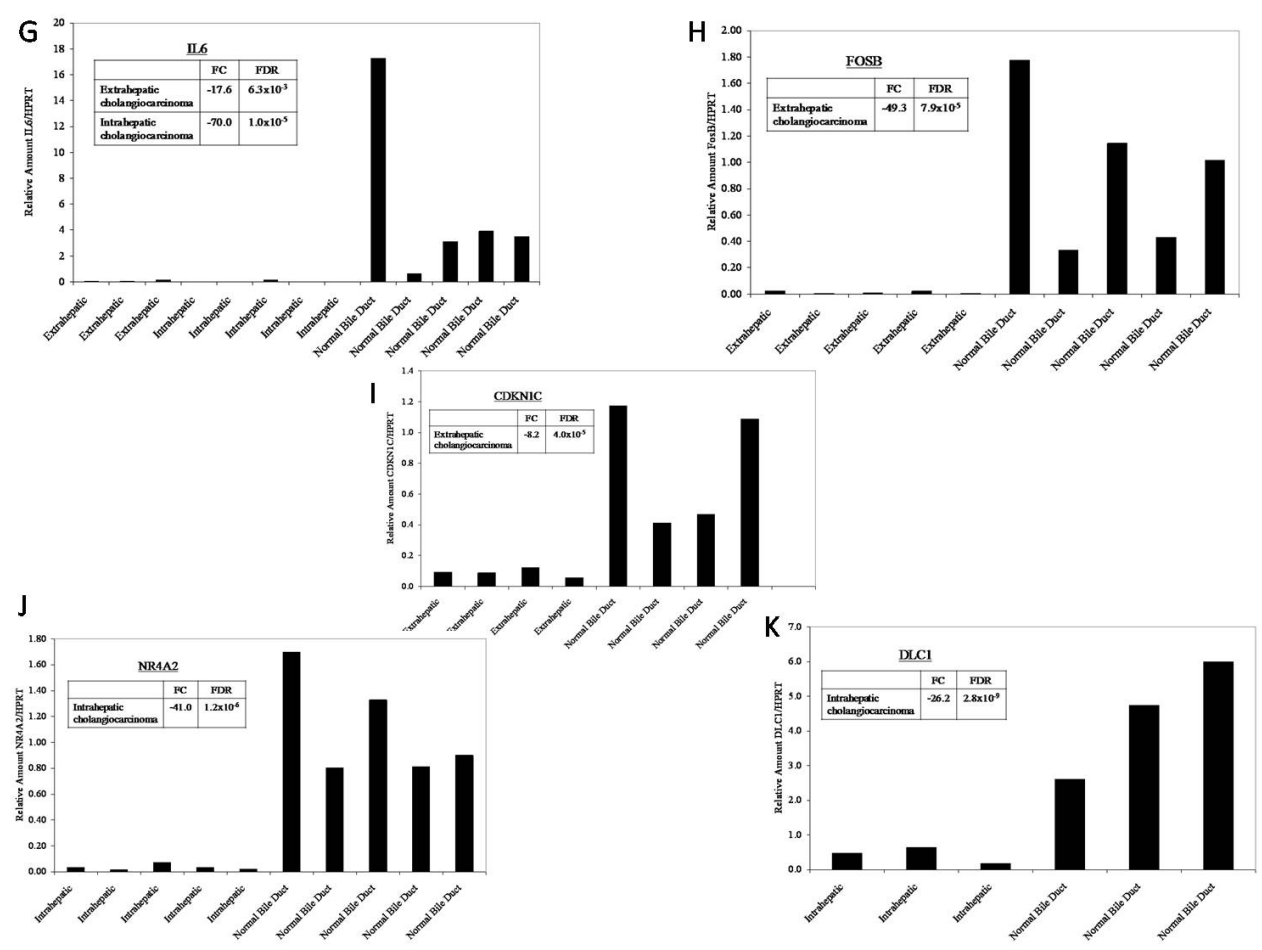
**Real-Time PCR Based Validation of Gene Expression Findings**. To confirm the gene expression changes in biliary tract cancers identified on microarray analysis, selected genes were tested in tumor and control specimens by RT PCR and normalized to *HRPT *which is similarly expressed in tumors and normal biliary epithelia. Results are shown for (g) *IL6*, (h) *FOSB*, (i) *CDKN1C*, (j) *NR4A2*, and (k) *DLC*.

In addition, to externally validate our data, selected differentially expressed genes were measured for transcript levels in biliary carcinoma specimens and in normal biliary epithelial controls using quantitative reverse transcriptase PCR. We assayed 11 genes with differing biologic functions and involvement in diverse molecular pathways but with known importance in carcinogenesis. These included genes which were overexpressed in EHC (*SRDA21, STAT1, UBD, TYMS*), underexpressed in EHC (*FOSB*, *CDKN1C*, *IL6*), overexpressed in IHC (*SRDA21, STAT1, UBD, TYMS*), underexpressed in IHC (*DLC1*, *NR4A2*, *IL6*), and overexpressed in GBC (*UBD*, *TYMS, CDC2, CCNB2*). PCR data was normalized to *HPRT *which was expressed at similar levels in both the cancerous and the control biliary epithelium (not shown). Results are shown in Figures (3a–f, 4g–k) and, for each gene tested, confirm the Affymetrix U133A gene expression array data. The array-based CGH results were internally validated by correlation of the X chromosome copy number with patient gender.

### Correlation of Gene Expression Profiles with Clinicopathologic Features

To determine whether certain clinicopathologic features are associated with specific gene expression changes in biliary carcinomas, we performed over-representation analyses by determining whether certain functional gene categories were over-represented among the top 100 ranking genes (by FDR) with altered expressing in patients with specific clinicopathologic features. Altered expression of genes associated with functional categories related to ribosomal structure, cellular and protein biosynthesis and cellular metabolism were significantly associated with high grade tumors (See additional file 8). Similarly, a strong correlation could be made between vascular invasion and mutated expression of genes involved with electron transport and metabolism (See additional file 9). Perineural invasion was correlated with altered expression of genes in the functional categories associated with mitochondrial structure and electron transport (See additional file 10). There was no significant association between gene expression patterns and lymph node invasion. Similarly, we did not find a significant correlation between functional gene category over-representation and survival.

## Discussion

The molecular pathogenesis of biliary tract cancers is poorly understood. By performing immunohistochemical analysis of more than 125 surgically resected cases of biliary tract carcinoma, we have previously shown altered cell cycle regulatory protein expression in biliary tact cancers [13]. Our current findings also show mutated expression of a large number of cell cycle regulators including *UBD*, *BCL2L2*, *CDC2*, *MCM2*, and *CDKN1C *in all subtypes. Similarly, Kang et al. [15] found that expression of G1-S modulators were commonly mutated in 42 cases of IHC. Total loss of p16, p27, and Rb were detected at rates of in 36%, 31%, 12%, respectively, in cancer specimens. Furthermore, in the above study, even in 7 of 13 cases of biliary dysplasia, without frank carcinoma, abnormal expression of p53, cyclin D1 or p16 was detected. Kim et al. [16] reported that the mutation of the p53, p16, and K-ras genes occurred at rates of 36%, 31% and 20%, respectively, in GBC. A further finding of the above study was that 100% of GBCs and 80% of adenomas displayed loss of heterozygosity at a minimum of one locus which is consistent with our CGH results. Chang et al. [17] studied loss of heterozygosity in 32 cases of GBC and 11 cases of dysplasia. Loss of one allele was identified on chromosomes 5q (55%) and 17p (40%) in the dysplastic cases and on chromosomes 3p (52%), 5q (66%), 9p (52%), and 17p (58%) in the carcinomas. Loss of heterozygosity on multiple chromosomes was significantly more frequent in patients with metastatic disease than in cases without metastases. In the current report, we similarly found that segments of 3p and 9p were commonly deleted across all subtypes of biliary cancers. However, we additionally discovered that segments of 6q, 8p, and 14q were commonly deleted across subtypes of biliary cancers

There is increasing evidence that overexpression of tyrosine kinase growth factor receptors such as ErbB-2, epidermal growth factor receptor (EGFR), and Met play important roles in the development of biliary tract carcinomas. Nakasawa et al. [18] studied tyrosine kinase receptor proteins expression by in 221 biliary tract carcinomas and found that overexpression of ErbB-2 was found in 16% of carcinomas of the gallbladder and a slightly lower percentage of extrahepatic bile duct tumors. ErbB-2 gene amplification was present in 79% of cases. Overexpression of EGFR was found in 8% of tumors and was also associated with a high frequency of gene amplification (77%). Met overexpression was most frequent in IHC (21.4%) but was not associated with gene amplification. Microsatellite instability also appears to be a critical factor in selected cases of biliary carcinogenesis. Roa et al. [19] performed microsatellite analysis on 59 frozen GBC specimens using 13 different markers. They found evidence of microsatellite instability in equal proportions in early and late cancers, and it was also found in premalignant lesions, indicating that inactivation of mismatch repair genes occurs early in gallbladder carcinogenesis.

In addition to finding that a large proportion of differentially expressed genes in this study involved in cell cycle regulation and apoptosis, we also discovered a disproportionate number of mutated genes that control transcriptional regulation, RNA procession, cellular signaling, or are involved with cytoskeletal structure, extracellular matrix, and cellular adhesion. Differentially expressed genes involved with transcriptional regulation include *STAT1*, *NARG1*, *HOXC6*, and *MMP11*. Important genes involved with signal transduction with altered expression include *CXCL5*, *ECT2*, *GPRC5A*, *MELK*, and *CKS2*. Dysregulated genes involved with cytoskeleton, extracellular matrix and cellular adhesion include *ITGA7*, *LAMB3*, *CECAM5*, *KRT6B*, and *CLDN18*.

The findings of the present study will serve as a resource for other investigators in this area as we have indentified many potential targets for therapeutic intervention. As an example, we found that *TYMS*, which encodes an enzyme that catalyzes 5-fluorouracil, was overexpressed 7.2 – 26.0-fold depending on biliary cancer subtype. *TYMS *expression is correlated inversely with clinical response to 5-fluorouracil-based chemotherapy and the overexpression may explain the futility of 5-fluorouracil-based chemotherapy for biliary carcinomas [20].

We also found that a number of genes in the ubiquitin pathway had altered expression in each cancer subtypes. For example, more than 20 ubiquitin-related genes had significantly altered expression IHC. In GBC, *UBD *was overexpressed more than 200-fold and *UBE2C *was overexpressed nearly 15-fold. Ubiquitin and ubiquitin-like proteins are signaling messengers that regulate a variety of cellular processes including cell proliferation, cell cycle regulation, DNA repair, and apoptosis. There is accumulating evidence that deregulation of this pathway as a result of mutations or altered expression of ubiquitylating or de-ubiquitylating enzymes as well as of Ub-binding proteins affect crucial mediators of these functions and are underlie the pathogenesis of several human malignancies [21]. A variety of inhibitors of the ubiquitin system are currently being experimentally tested in clinical trials with promising early results [22]. These data suggests these inhibitors may have applicability as adjuvants in treating patients with biliary tract carcinomas.

Another promising target uncovered in this report is *STAT-1 *which was overexpressed nearly 9-fold in cases of cholangiocarcinoma. The Signal Transducers and Activator of Transcription (STAT) proteins regulate many aspects of cell growth, survival and differentiation. The transcription factors of this family are activated by the Janus Kinase JAK and dysregulation of this pathway has been observed in primary tumors and leads to increased angiogenesis, metastases, enhanced survival of tumors, and immunosuppression [23,24]. A number of JAK/STAT pathway inhibitors are being tested in pre-clinical studies and their application to cancers of the biliary tract may prove promising [25].

## Conclusion

Both gene expression and CGH data support an overlapping pathogenetic mechanism for all subsets of biliary tract cancers. However, exceptional diversity of mutational findings between individual patient specimens is also apparent. Functional over-representation analysis revealed a significant association between altered expression of genes involved with regulation of cellular metabolism and biosynthesis and high pathologic grade. Vascular invasion was associated with mutated expression of genes involved with electron transport and cellular metabolism. CGH analysis revealed that short segments of chromosomes 1p, 3p, 6q, 8p, 9p, and 14q were commonly deleted across all cancer subtypes while commonly amplified regions included segments of 1q, 3q, 5p, 7p, 7q, 8q, and 20q. The data also offer opportunities to uncover potential targets for experimental therapeutics.

## Competing interests

The authors declare that they have no competing interests.

## Authors' contributions

GM carried out the conception and design, acquisition, analysis, and interpretation of data, drafting of manuscript, critical review, and final approval. NDS contributed in the conception and design, analysis and interpretation of data, critical review, and final approval. DD contributed in the acquisition of data, and final approval. MD contributed in the conception and design, critical review, and final approval. RPD contributed in the conception and design, critical review, and final approval. PJA contributed in the conception and design, critical review, and final approval. BS contributed in the conception and design, critical review, and final approval. YF contributed in the conception and design, critical review, and final approval. LHB contributed in the conception and design, critical review, and final approval. DSK contributed in the conception and design, analysis and interpretation of data, critical review, and final approval. WRJ carried out the conception and design, analysis and interpretation of data, drafting of manuscript, critical review, and final approval. All authors have read and approved the final manuscript.

## Supplementary Material

Additional File 1 **Gene Expression Changes in Extrahepatic Cholangiocarcinoma.** Click here for file

Additional File 2 **Gene Expression Changes in Intrahepatic Cholangiocarcinoma.** Click here for file

Additional File 3 **Gene Expression Changes in Gallbladder Cancer.** Click here for file

Additional File 4 **Commonly Differentially Expressed Genes in All Biliary Cancer Subtypes.** Click here for file

Additional File 5 **Gene Expression Changes in Unstable Genomic Regions for Extrahepatic Cholangiocarcinoma.** Click here for file

Additional File 6 **Gene Expression Changes in Unstable Genomic Regions for Intrahepatic Cholangiocarcinoma.** Click here for file

Additional File 7 **Gene Expression Changes in Unstable Genomic Regions for Gallbladder Cancer.** Click here for file

Additional File 8 **Over-representation Analysis – Tumor differentiation.** Click here for file

Additional File 9 **Over-representation Analysis – Vascular Invasion.** Click here for file

Additional File 10 **Over-representation Analysis – Perineural Invasion.** Click here for file
